# Enhancing Mass Lesion of the Sphenoid: Atypical Presentation of Ongoing Pneumatization

**DOI:** 10.1155/2016/2862010

**Published:** 2016-12-12

**Authors:** Deepak Vallabhaneni, Anthony Mohamed, Zain Badar, Rajiv Mangla

**Affiliations:** Department of Radiology, SUNY Upstate Medical University, Syracuse, NY 13210, USA

## Abstract

Sinus pneumatization is a complex variable process that begins in early life and continues for many years. We present a case of a 6-year-old boy with progressive headaches and neurologic symptoms suggestive of intracranial pathology. The presence of enhancing tissue within the sphenoid sinus created a diagnostic dilemma which leads to a transsphenoidal biopsy. Knowledge of imaging characteristics associated with incomplete pneumatization can help differentiate it from more ominous skull base pathology and prevent unnecessary testing. We describe four-year imaging follow-up in a patient with incomplete pneumatization of the sphenoid sinus presenting as an enhancing mass lesion with subsequent follow-up imaging demonstrating gradual regression and increased aeration of the sphenoid sinus.

## 1. Introduction

Sinus pneumatization is a prolonged developmental process beginning in utero and extending into early adulthood [1]. Early in life, the red marrow within the sphenoid typically undergoes fatty conversion with subsequent aeration of the sinus with extension of respiratory mucosa. This occurs most rapidly from 1 to 5 years of age with continued expansion [2]. However, pneumatization is highly variable, both developmentally and anatomically [3, 4]. Unfamiliarity with these imaging findings may result in confusion for radiologists and clinicians [5]. Early recognition of incomplete or arrested pneumatization can prevent unnecessary testing, treatment, and emotional distress.

## 2. History and Presentation

A 10-year-old male, who initially presented at age 6 with frontal headaches, complained of increasing headache frequency over the preceding months, occurring up to 3-4 times per week. He also experienced associated photophobia and phonophobia. His family history is notable for migraines. The patients' symptoms are initially managed conservatively.

The patient was brought to the emergency department by his father after developing acute loss of vision in his right eye with right upper extremity numbness. He again complained of headache at this time. The patient noted that his vision had returned to normal by the time he had arrived at the hospital. Given the acuity of his symptoms with focal neurological deficits, the patient underwent an MRI and MRA of the head, as well as an MRI of the orbits as part of his diagnostic workup.

MR imaging demonstrated an enhancing lobular lesion with T2/FLAIR hyperintensity and T1 isointensity in the sphenoid with extension laterally into the greater wing of the sphenoid bilaterally. These findings were concerning for a neoplastic process which prompted endoscopic transsphenoidal biopsy. Tissue sampling revealed the presence of benign tissue composed of fragments of respiratory epithelium, fibrous tissue with scattered chronic inflammation, and hemorrhage with no neoplastic elements.

Over the following few years, his symptoms were treated as a chronic migraine disorder but the patient was unresponsive to most front line migraine treatments. Following additional unremarkable neurological tests, further diagnostic imaging was performed over the course of the following four years. Subsequent imaging demonstrated ongoing gradual decrease in the size of the enhancing tissue in the sphenoid sinus with increasing aeration. The diagnosis of incomplete pneumatization of the sphenoid sinus was then made.

## 3. Discussion

Pneumatization of the sphenoid sinus is a complex process, beginning with marrow conversion in the presphenoid before progressing posteriorly to the basisphenoid plate. This progression continues inferiorly and posteriorly adjacent to the clivus [6]. The timeline of this process varies considerably, with one study demonstrating 89% of patients having presphenoid fatty marrow conversion by age 2 [7]. Another study showed 48% of children within the ages of 4–6 years with persisting T1 hyperintensity of the sphenoid sinus [8]. By age 6, 85% of children have some signs of initial anterior sphenoid aeration [8].

The structural anatomy of the sphenoid sinus is highly variable. There are four main structural configurations of sphenoid sinuses. The sinus may be small or missing (type I); the position of the posterior sphenoid wall may be anterior to the anterior wall of the sella turcica (type II), posterior to the posterior wall of the sella turcica (type IV), or interposed (type III) [4].

There are multiple hypotheses postulating the mechanism of pneumatization. One such theory is the invasive tissue hypothesis. Mucous epithelium invades adjacent osseous structures, allowing mechanical stability of the complex sinus system [9]. Triggers of this complex process may include decreasing oxygen tension and temperature changes which allow hematopoietic red marrow to convert into fatty marrow prior to epithelial cells becoming involved [10]. The ethmoidal expansion theory states that aeration within the sphenoid sinus is secondary to expansion of ethmoid air cells into the adjacent sinuses [11]. Additional theories have been proposed; however none of these have fully elucidated the exact mechanism [12].

**Figure 1:**
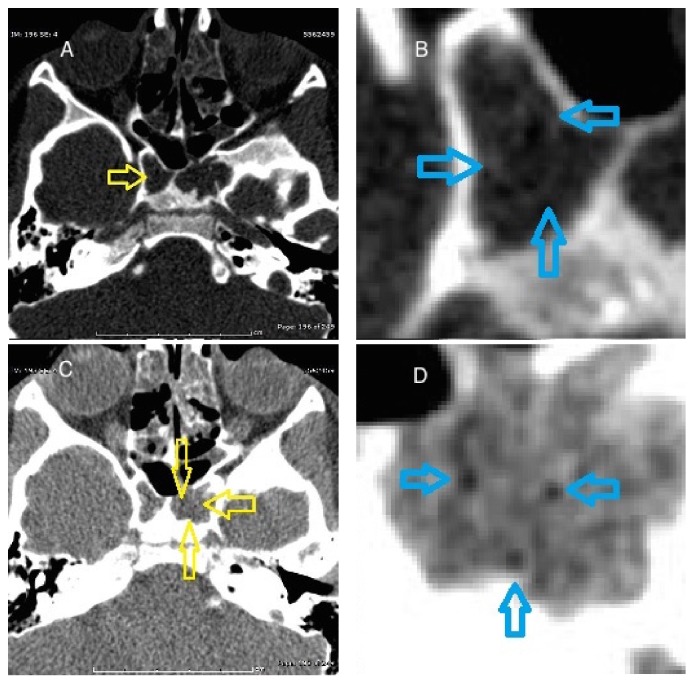
Axial unenhanced CT images through the sphenoid sinus. (A) Curvilinear calcifications (yellow open arrow) are seen in the sphenoid sinus when viewed utilizing bone algorithm. (B) Magnified views demonstrate additional curvilinear calcifications (blue open arrows). (C) When viewed utilizing lung algorithm, there are multiple small foci of fat identified (yellow open arrows). (D) Magnified views more clearly demonstrate these foci (blue open arrows).

Fatty marrow may persist adjacent to a partially pneumatized sinus and may appear mass-like, creating a challenging diagnostic dilemma [5]. Delayed, incomplete, or arrested pneumatization presents as a nonexpansile lesion occurring at a site of expected sinus pneumatization or accessory pneumatization. The internal contents will demonstrate curvilinear calcifications and foci of fat. The margins will be thin and sclerotic while respecting the borders of the adjacent exiting nerves.  Figure 1 demonstrates the CT findings associated with our lesion which fulfill these criteria [5].

**Figure 2:**
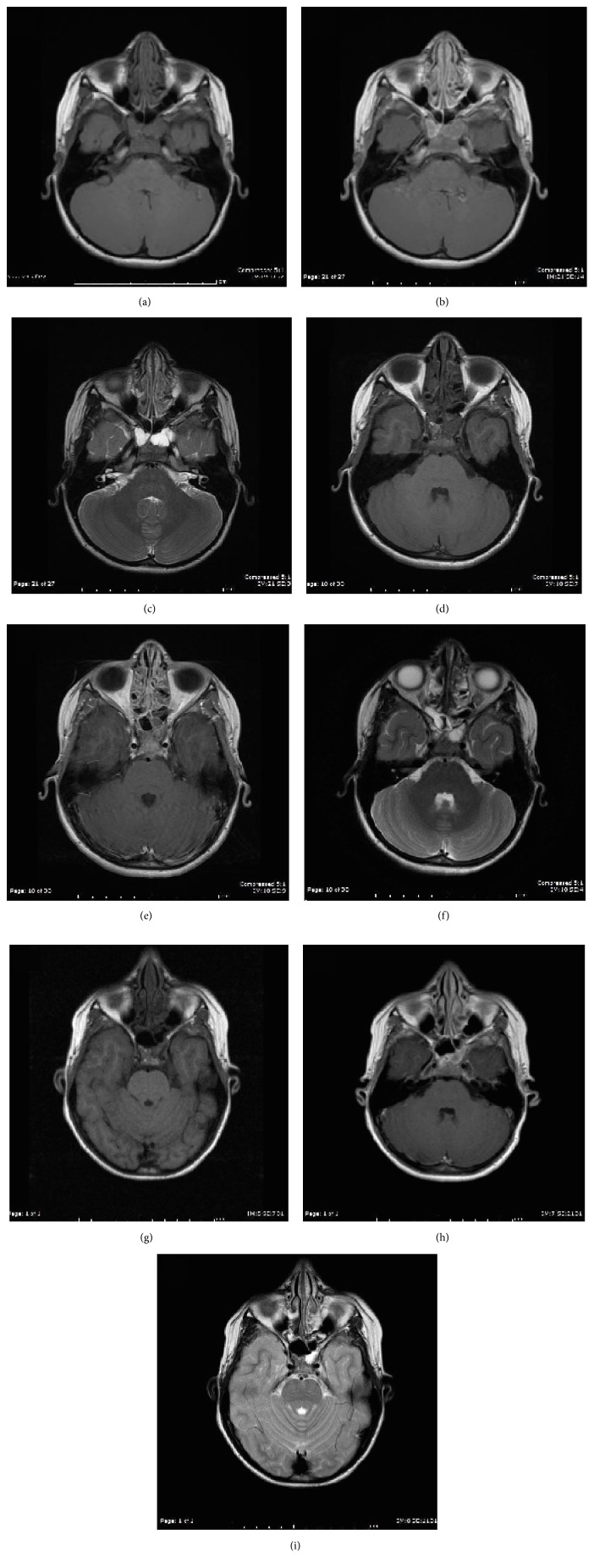
MR T1 (a), T1 postcontrast (b), and T2 (c) weighted images through the sphenoid sinus at age 6. There is T1 isointense and T2 hyperintense soft tissue within the sphenoid sinus, which shows postcontrast enhancement. MR T1 (d), T1 postcontrast (e), and T2 (f) weighted images through the sphenoid sinus at age 8. There is overall decreased soft tissue mass within the sphenoid sinus with increased T1 signal intensity, likely related to ongoing fatty marrow conversion. MR T1 (g), T1 postcontrast (h), and T2 (i) weighted images through the sphenoid sinus at age 10. There is pneumatization within the anterior sphenoid sinus with a small amount of residual soft tissue noted posteriorly.

The clinical picture was further complicated by the presence of postcontrast enhancement within the lesion on MRI (Figure 2). Persistent marrow is typically associated with little to no postcontrast enhancement on MR; however, evaluation for enhancement can be difficult as the fatty components are typically T1 hyperintense and postcontrast fat suppressed images may not be obtained. Our patient demonstrated multiple small foci of fat which are identified on CT and MRI, however containing a prominent soft tissue component. Follow-up MR imaging obtained at ages 8 and 10 demonstrates increasing T1 signal intensity and increased aeration of the sinus over time, which is presumed to represent ongoing fatty marrow conversion followed by pneumatization of the sinus. Additionally, well circumscribed lesions are more likely to be related to ongoing pneumatization, while those with evidence of bone erosion point towards an alternative diagnosis [10].

The differential diagnosis for an enhancing skull base lesion is broad. One notable differential diagnosis is fibrous dysplasia; characteristic CT findings of ground glass marrow can aid with this diagnosis. Other important differential diagnoses include ossifying fibroma, chordoma, and chondrosarcoma. Both ossifying fibroma and arrested pneumatization are well circumscribed; ossifying fibromas tend to be more expansile and do not commonly occur in the skull base. Chordomas and chondrosarcomas also tend to be expansile and destructive while lacking central fat [5]. An intraosseous lipoma is also included in the differential based on similar characteristics such as internal fatty matrix and T1 hyperintensity, however tending to be expansile and unlikely in the skull base [13]. A full differential diagnosis includes intraosseous hemangioma, hamartoma, and enchondroma in addition to those listed above [14].

Clinicians and diagnostic radiologists should be aware of the process of paranasal sinus development and its corresponding imaging characteristics. Sinus structure and aeration are highly variable in children creating difficultly in differentiating normal variation from worrisome pathology. Familiarity with the normal patterns of sinus development is therefore crucial in recognizing this entity. Although the differential for enhancing skull based lesions is broad, identifying the characteristic findings associated with ongoing or arrested pneumatization, such as internal fatty components, curvilinear calcifications, and nonexpansile well circumscribed borders, can allow for confident diagnoses and avoid unnecessary intervention.
